# Optimization of the Obtaining of Cellulose Nanocrystals from *Agave tequilana* Weber Var. Azul Bagasse by Acid Hydrolysis

**DOI:** 10.3390/nano11020520

**Published:** 2021-02-18

**Authors:** Manuel Alberto Gallardo-Sánchez, Tania Diaz-Vidal, Alejandra Berenice Navarro-Hermosillo, Edgar Benjamin Figueroa-Ochoa, Rogelio Ramirez Casillas, José Anzaldo Hernández, Luis Carlos Rosales-Rivera, J. Felix Armando Soltero Martínez, Salvador García Enríquez, Emma Rebeca Macías-Balleza

**Affiliations:** 1 Departamento de Ingeniería de Proyectos, Centro Universitario de Ciencias Exactas e Ingenierías, Universidad de Guadalajara, Guadalajara C.P. 44430, Mexico; manuel.gallardo@academicos.udg.mx (M.A.G.-S.); alenicenavarro@gmail.com (A.B.N.-H.); 2 Departamento de Ingeniería Química, Centro Universitario de Ciencias Exactas e Ingenierías, Universidad de Guadalajara, Guadalajara C.P. 44430, Mexico; taniadzv@gmail.com (T.D.-V.); carlos.rosales@academicos.udg.mx (L.C.R.-R.); jfasm@hotmail.com (J.F.A.S.M.); 3 Departamento de Química, Centro Universitario de Ciencias Exactas e Ingenierías, Universidad de Guadalajara, Guadalajara C.P. 44430, Mexico; benjamin.figueroa@academicos.udg.mx; 4 Departamento de Madera Celulosa y Papel, Centro Universitario de Ciencias Exactas e Ingenierías, Universidad de Guadalajara, Zapopan C.P. 45020, Mexico; roramire@gmail.com (R.R.C.); j.anzaldo@academicos.udg.mx (J.A.H.); salgaren@hotmail.com (S.G.E.)

**Keywords:** *Agave tequilana*, bagasse, cellulose nanocrystals, acid hydrolysis, AFM, factorial design

## Abstract

A multilevel factorial design of 2^3^ with 12 experiments was developed for the preparation of cellulose nanocrystals (CNC) from *Agave tequilana* Weber var. Azul bagasse, an agro-industrial waste from tequila production. The studied parameters were acid type (H_2_SO_4_ and HCl), acid concentration (60 and 65 wt% for H_2_SO_4_, 2 and 8N for HCl) temperature (40 and 60 °C for H_2_SO_4_, 50 and 90 °C for HCl), and hydrolysis time (40, 55 and 70 min for H_2_SO_4_; and 30, 115 and 200 min for HCl). The obtained CNC were physical and chemically characterized using dynamic light scattering (DLS), atomic force microscopy (AFM), Fourier-transform infrared spectroscopy (FT-IR), X-ray photoelectron spectroscopy (XPS), and X-ray diffraction (XDR) techniques. The maximum CNC yield was 90 and 96% for HCL and H_2_SO_4_, respectively, and the crystallinity values ranged from 88–91%. The size and morphology of *A. tequilana* CNC strongly depends on the acid type and hydrolysis time. The shortest CNC obtained with H_2_SO_4_ (65 wt%, 40 °C, and 70 min) had a length of 137 ± 68 nm, width 33 ± 7 nm, and height 9.1 nm, whereas the shortest CNC obtained with HCl (2 N, 50 °C and 30 min) had a length of 216 ± 73 nm, width 69 ± 17 nm, and height 8.9 nm. In general, the obtained CNC had an ellipsoidal shape, whereas CNC prepared from H_2_SO_4_ were shorter and thinner than those obtained with HCl. The total sulfate group content of CNC obtained with H_2_SO_4_ increased with time, temperature, and acid concentration, exhibiting an exponential behavior of $C_{SG}=ae^{bt}$.

## 1. Introduction

Cellulose nanocrystals (CNC) are highly crystalline nanoparticles shaped like an elongated bar, with diameters between 2–20 nm, and lengths of <500 nm [1,2]. CNC have desirable physical, chemical, and mechanical properties, such as an elastic modulus around 150 GPa, which is greater than the elastic modulus of glass fibers (85 GPa) and aramid (65 GPa) [3,4,5], high tensile strength (7500 MPa), high stiffness (Young’s modulus ≥140 GPa), a high surface area, high aspect ratio, an abundance of surface hydroxyl groups available for chemical functionalization, and full biodegradability, due to its natural origin [1,2,3,4,6]. Therefore, CNC have been applied in diverse fields such as medicine [2], catalysis [7], biosensing [8], and as reinforcing materials to improve the mechanical properties of composites in multiple applications, such as plastics, ceramics, concrete, etc. [9]. Recently, it has been observed that the reinforcement of cementitious materials with cellulose microcrystals (CMC) and CNC at concentrations <2 wt.% improve the flexural and compressive strength between 20 and 50% of the final material [10,11,12], and the tensile strength and Young’s modulus of reinforced poly(3-hydroxybutyrate-co-3-hydroxyvalerate) (PHBV) with 12% of CNC-H were improved by 175 and 300%, respectively [13].

Typically, CNC are obtained through the removal of the amorphous cellulose regions by chemical means [3,6]. The prior purified cellulose material is subjected to hydrolysis with a strong acid under strictly controlled conditions, followed by dialysis in water and successive washes to remove the free acid. Usually, an extra step with ultrasound and filtration is considered to separate residues and ensure a stable colloidal suspension [5,14,15]. The amorphous regions in the cellulose chains are more susceptible to acid hydrolysis than crystalline regions, thus the breakdown of glycosidic bonds is facilitated, releasing the individual crystals. H_2_SO_4_ and HCl are frequently used for the preparation of CNC, however phosphoric and hydrobromic acids have been regularly employed [16]. The most studied parameters for acid extractions with H_2_SO_4_ include hydrolysis time from 40–70 min, temperatures ranging from 40–60 °C and acid concentrations between 60–65 wt% [15,16,17,18,19,20,21]. In the case of HCl, the most used conditions are 30–200 min, 60–110 °C, and concentrations from 2–8 N [13,22,23,24].

*Agave tequilana* Weber var. Azul is the main resource in the tequila industry. To generate 1 L of tequila, *A. tequilana* heads are cooked and crushed to extract the sugars, to finally generate approximately 1.4 kg of bagasse in wet weight. According to the Tequila Regulatory Council, 523,600 tons of bagasse from all the *A. tequilana* varieties were obtained in the process of tequila generation solely in 2020, of which 319,620 tons corresponded to *A. tequilana* Weber var. Azul [25]. The excess bagasse creates an enormous environmental impact in terms of agro-waste management and disposal [26]. Extensive effort has focused on the comprehensive exploitation of *A. tequilana* bagasse in the last decade. Many studies point to the high cellulose content of *A. tequilana* bagasse, close to 80% [27,28], as the main source for the production of high-added value sub-products, such as cellulose nanocrystals [29].

Depending on the cellulose raw material pretreatment and hydrolysis process, CNC with tunable properties can be obtained [30]. CNC extracted with HCl show poor colloidal stability, whereas CNC produced with H_2_SO_4_ form highly stable colloidal dispersions due to electrostatic repulsion caused by negatively charged sulfate ester groups on their surface [31]. Increasing temperatures and reaction times have been associated with smaller nanocrystal sizes; however, severe reaction conditions can reduce the yield and shorten the crystal size [19,32]. Thus, a comprehensive study analyzing acid concentration and determining the best operational output is mandatory to universally optimize the obtaining of CNC. In this sense, the design of a factorial experiment can be employed to optimize the process of CNC production from *A. tequilana* bagasse. The design aims to determine if the principal factors are statistically significant based on a null hypothesis test, with a confidence value of *p* < 0.05 [22,33]. Compared to one-factor-at-a-time experiments, factorial designs allow the detection and study of interactions in a more efficient way [34]. This technique has already been employed to optimize the isolation and production of microcrystalline and nanocrystalline cellulose from recycled wood pulp [35], *Picea abies* [36], and *Acacia farnesiana L. Willd* bagasse [37] with a reduced number of experiments, time, and cost.

In the present work, two multilevel factorial 2^3^ experimental designs were selected to optimize the process of CNC isolation from *A. tequilana* by acid hydrolysis with H_2_SO_4_ and HCl. The studied factors were acid concentration, reaction time, and temperature. To determine the best and statistically significant operational conditions, the CNC dimensions of length (L), height (H), and width (W) were measured from AFM images, while diameter (d) of dispersed particles were measured from DLS. The reaction yields of all studied parameters were also compared. Finally, the obtained CNC were chemically characterized with XPS, FTIR, and XRD.

## 2. Materials and Methods

### 2.1. Materials

Bagasse from *A. tequilana* Weber var. Azul was kindly donated by Mundo Agave (Tequila, Jalisco, Mexico). Sulfuric acid (H_2_SO_4_, 97 wt%) and hydrochloric acid (HCl, 37 wt%) were obtained from Golden Bell (Mexico). Dialysis membranes Spectra/Por 4 MWCO 12-14 KDa were purchased from Thomas-Scientific (Swedesboro, NJ, USA). Filters were provided by Thermo Fisher Scientific (Waltham, MA, USA). All solutions were prepared using milli-Q water with a resistivity of 18.2 MOhm x cm at 25 °C.

### 2.2. Experimental Design

Two multilevel factorial designs of 2^3^ were developed via STATGRAPHICS Centurion XVI (The Plains, VA, USA) to investigate the effect of acid concentration, hydrolysis time, and temperature on the final length measured using DLS and AFM, and yield of CNC produced from *A. tequilana* Weber var. azul. The experimental factor details are described in Table 1 and the detailed list of controllable process factors is shown in Table 2. CNC obtained by H_2_SO_4_ and HCL hydrolysis will be denoted as CNC-S and CNC-H, respectively.

### 2.3. Preparation of Cellulose Nanocrystals

Soluble-grade cellulose pulp from *A. tequilana* was obtained according to Gallardo-Sánchez et al. [27]. Briefly, soluble-grade cellulose was ground with an IKA MF 10 mill (Staufen, Germany) until particles passing through the 0.5 mm sieve were obtained. The hydrolysis was carried out with variable conditions of concentration, temperature, and time (Table 2), with 12 different conditions for each acid. The acidic solutions were prepared and poured into a three-necked reactor with a capacity of 1 L, and then the solution was heated in an isothermal Haake bath C1 (Vreden, Germany), with a mechanical stirrer RZR 50 Caframo (Ontario, Canada) at 300 rpm. Next, 20 g of soluble-grade cellulose (dry base) were added at the corresponding time. Ratios of 10:1 (*v*/*w*) and 30:1 (*v*/*w*) were used for the hydrolysis with H_2_SO_4_ and HCl, respectively.

After the hydrolysis reaction, the CNC solution was poured into deionized water at 4 °C, centrifuged (4696 rcf, for 45 min), and the precipitates were dialyzed in deionized water (12-14 KDa) at room temperature until the pH reached a value of 5.5. The water was replaced every 12 h. Afterwards, the CNC were sonicated for 8 min in an Elma ultrasound bath model TI-H-15 (Singen, Germany), filtered through 2, 1.5, and 1 µm filters, and stored at 4 °C until further use.

### 2.4. Determination of Sulfate Groups in CNC-S

The residual concentration of sulfate groups on the CNC-S surface (mmol/kg) was measured by conductometric titration, as reported for CNC from huizache wood [32]. A Thermo Fisher Scientific Orion Star A211 potentiometer (Waltham, MA, USA) was used. The titration of sulfate groups ($C_{SG}$) was performed using 0.05 N NaOH and 30 mL of a CNC-S solution in 150 mL of deionized water in a three-neck flask. The analysis of the samples started by adding 0.5–1 mL of NaOH to the CNC suspension at a constant stirring. pH and conductivity values were recorded until no further variations were detected. *V*_2_ was determined from the intercept of two slopes obtained from the conductivity vs NaOH volume plot. The sulfate groups were calculated as follows:

(1) $$C_{SG}=Ct*\frac{V_{2}}{m}$$

where *C_SG_* is the total sulfate groups content in mmol/kg, *Ct* is the NaOH concentration (in this case, 0.05 N), *V*_2_ is the NaOH consumed volume in mL, and *m* is the dried mass of CNC-S in *g*.

### 2.5. X-ray Photoelectron Spectroscopy (XPS)

XPS, which is a quantitative analysis method, measures the energy of excited electrons with an X-ray beam, at the lowest energy levels. This technique is performed within the first 10 nm of the surface and allows to identify the elemental composition at atomic concentrations greater than 0.1% (except for H and He), with an error <10% [38]. Monochromatic radiation (Al kα radiation, hν = 1486.7 eV) for XPS was generated using a SPECS GmbH XR50M twin anode X-ray source and a Phoibos 150 spectrometer with one-dimensional detector 1D-DLD (Berlin, Germany). XPS spectra were taken at an electro emission angle of 90°. The calibration of binding energy was realized with C1s peak at 284.8 eV. The XPS survey and high resolution spectra of soluble grade cellulose pulp and CNC were recorded. The analyses were carried out with a pass energy of 30 eV and 2 scans for survey, and 15 eV and 20 scans for high resolutions spectra. The elements O, S, and C were analyzed using the lines C1s, O1s, and S2p. Once the high resolution spectra were acquired, the component deconvolution and the area under the curve were obtained using the A-Analyzer software. Quantitative analyses were done after the subtraction of the baseline using the Shirley method.

The percentage of each element detected can be determined with Equation (2), where % *n_i_* is the atomic percentage of element *i*.

(2) $$\%n_{i}=100\;n_{i}/\sum n_{1}$$

*n_i_* is also determined by Equation (3), where, the values of each element must be considered.

(3) $$\%n_{i}=\frac{I_{ij}/\sigma_{ij}KE^{0.7}}{\sum (I_{ij}/\sigma_{ij}EK^{0.7})}$$

*KE* is the kinetic energy in eV, *σ_ij_* and *I_ij_* are the Scofield factor and the area under the curve, respectively, for the “*i*” element of “*j*” component [39].

### 2.6. Fourier-Transform Infrared Spectroscopy (FTIR)

FTIR measurements were performed with a Perkin-Elmer Spectrum GX (Waltham, MA, USA) in ATR mode. The spectra of powdered CNC were obtained in the transmittance mode by recording 16 scans at a resolution of 4 cm^−1^, and a frequency range from 4000 to 700 cm^−1^.

### 2.7. X-ray Diffraction (XRD)

CNC were analyzed with Malvern Instruments XRD Empyrean (Malvern, UK) with Cu-Kα radiation at 45 kV and a current of 40 mA. The incidence angle was set to 5–70° (step size of 0.2°). The percentage of crystallinity was calculated according to the Rietveld method [40], and the obtained data was analyzed with the University of Trento MAUD 2.1 software (Trento, Italy).

### 2.8. Atomic Force Microscope (AFM)

The morphology of CNC was characterized using Park Systems AFM NX10 (South Korea). CNC dispersions (0.005%, in water) were sonicated in an ultrasonic bath. Next, 10 mL of CNC dispersions were transferred to a metal microscope slide and air-dried. CNC images were obtained via tapping mode in the air using Budget Sensors Tap300-G probe (Izgrev, Sofia, Bulgaria). AC160TS-R3 micro silicon tips, coated with aluminum from Oxford Instruments (Abingdon, UK) were used for image recording. The amplitude and height of the samples were obtained from 1 μm × 1 μm images, and a resolution of 512/512 pixels/line. At least 30 CNC were measured and the AFM dimensions (L, H, and W) were obtained.

### 2.9. Dynamic Light Scattering (DLS) and Z-Potential

The CNC average size (d) and polydispersity index (PdI) was measured using dynamic light scattering (DLS) from Malvern Instruments Zetasizer Nano Series ZS90 (Malvern, UK). CNC solutions were first sonicated in an Elma P120H ultrasound bath (Singen, Germany), at a frequency of 40 kHz and 30% power, at room temperature, for 30 min. Subsequently, the second dilution of CNC was made (1:10 ratio) and sonicated again for 10 min. Between 6 and 18 scans were made for each sample, each scan being the average of 10 repetitions. DLS was used with a dispersant refractive index of 1.33 and absorption index of 0.01. Z-potential measurements were made using the same diluted samples prepared for DLS, with a disposable capillary cell (DTS1070).

## 3. Results

### 3.1. Yield and Factorial Design

CNC of *A. tequilana* bagasse were prepared using 24 experimental acid hydrolysis conditions (12 conditions with H_2_SO_4_ and 12 conditions with HCl) (Table 2). The maximum CNC yields obtained were 96% and 90% for experimental conditions E8S and E8H, respectively. The minimum CNC yields were observed with the experimental conditions E1S and E1H, with values of 4.2% and 4.5%, respectively. The standardized Pareto plots for CNC-S and CNC-H yield showed that any factor or interaction between factors was statistically not significant (Figure S1). The complete list of obtained yields for all experimental is shown in Tables S1 and S2 for HCl and H_2_SO_4_, respectively.

### 3.2. Determination of Sulfate Groups in CNC-S

**Figure 1:**
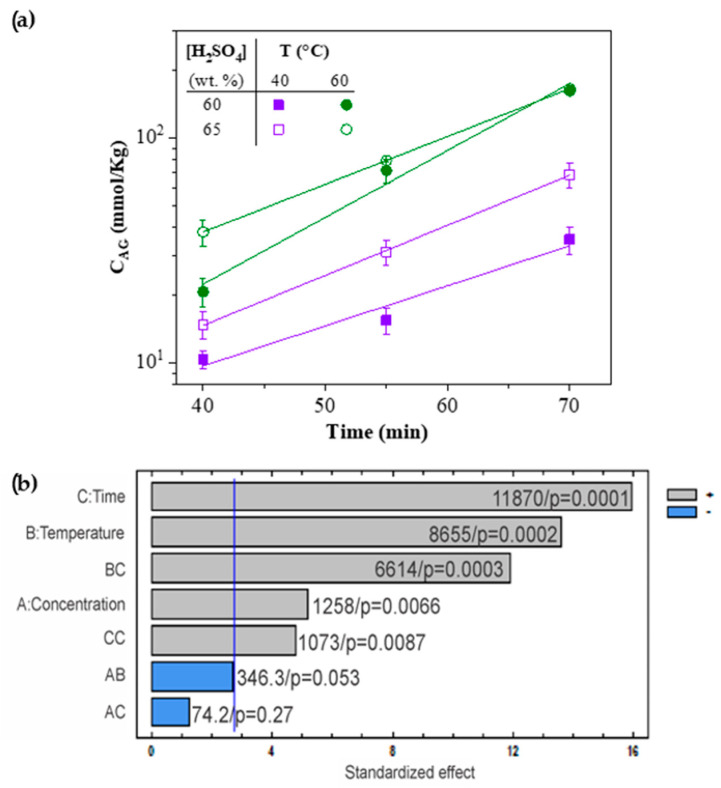
(**a**) Total sulfate groups content (*C_SG_*) as a function of time, for different temperatures, and H_2_SO_4_ concentrations. The lines represent the exponential equation: $C_{SG}=ae^{bt}$. (**b**) Standardized Pareto diagram for total sulfate groups present in CNC-S, the sum of squares, and the *p*-value are shown at the right side of each bar.

Figure 1a shows the total sulfate groups, i.e., the residual charge of H_2_SO_4_ on the CNC-S surface, as a function of hydrolysis time for all H_2_SO_4_ hydrolysis experiments. The total content of sulfate groups (*C_SG_*) increased with time, temperature, and acid concentration, exhibiting an exponential behavior of $C_{SG}=ae^{bt}$. The “a” and “b” equation values are shown in Table 3.

The principal factors and factor interactions with higher influence are shown in the Pareto diagram (Figure 1b). Positive effects indicate an increase from the minimum level to the maximum, while negative effects imply the opposite [41]. Concentration, time, and temperature are statistically significant factors (*p* < 0.05) contributing to the increase in *C_SG_*. The highest amount of sulfate groups was quantified for the experimental conditions E7S (162.0 mmol/kg) and E8S (166 mmol/kg). The complete list of total sulfate groups content is shown in Table S3. Our results are similar to those obtained for other sources, such as huizache wood (39.78 mmol/kg with 65% H_2_SO_4_, 45 °C and 45 min reaction, and 26.9 mmol/kg with 60% H_2_SO_4_, 55 °C and 65 min of reaction) [32]. Interactions BC (time and temperature) and CC (quadratic time) were also statistically significant (*p* < 0.05), and had a positive effect.

### 3.3. X-ray Photoelectron Spectroscopy (XPS)

**Figure 2:**
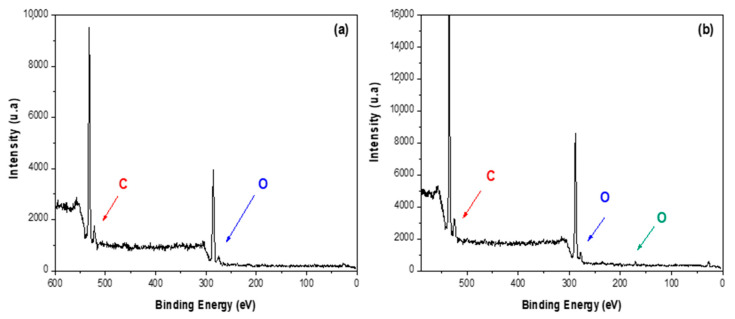
XPS spectrum of (**a**) soluble-grade cellulose pulp, and (**b**) CNC-S (E8S).

Soluble-grade cellulose pulp and CNC-S were analyzed by XPS before and after acid hydrolysis at the most severe condition tested (E8S). In the inspection spectrum of soluble-grade cellulose (Figure 2a) and CNC-S (Figure 2b), the main peaks are located at 285 and 532 eV, corresponding to C and O atoms, respectively. In the case of CNC-S, the presence of an S atom peak at 164 eV is observed, which is a typical feature of H_2_SO_4_ hydrolysis.

**Figure 3:**
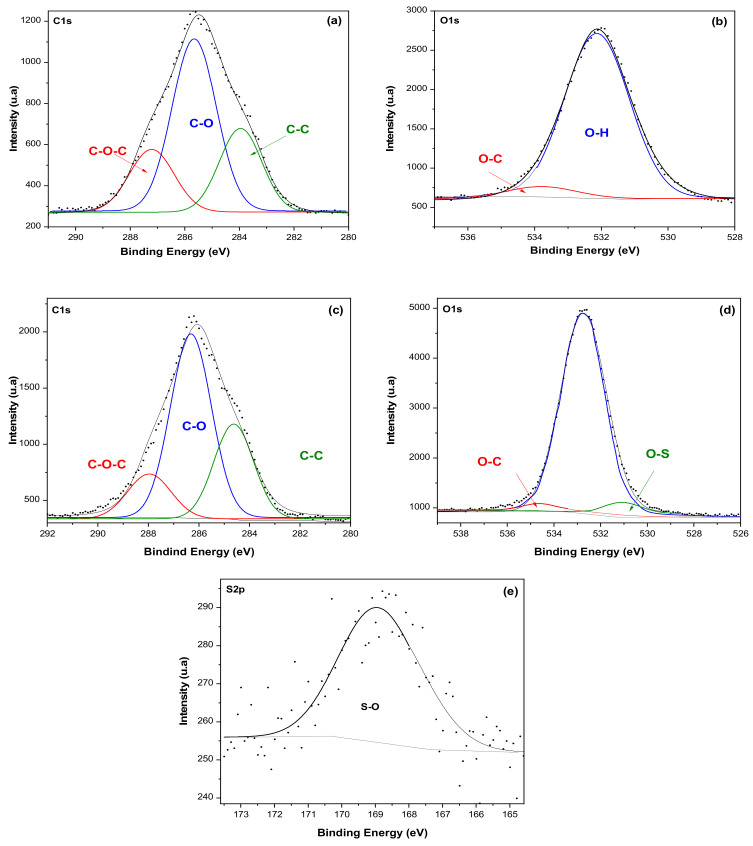
High resolution spectra for soluble-grade cellulose pulp: (**a**) C1s, (**b**) O1s, and CNC-S: (**c**) C1S, (**d**) O1s, and (**e**) S2p.

The deconvolution spectra were performed for C1s (Figure 3a), and O1S (Figure 3b) for soluble-grade cellulose, and C1s (Figure 3c), O1s (Figure 3d), and S2p (Figure 3e) for CNC-S. In the high resolution C1s spectra, the C signal is resolved into three distinctive peaks for both soluble cellulose and CNC-S, with bond energies at 283, 285, and, 288 eV, attributed to C-C, C-O, C-OH, and C-O-C bonds, respectively. In the high resolution O1s spectra, two peaks were observed at 532 and 533 eV, attributed to O-H and O-C bonds for soluble cellulose spectra. In the O1s spectra of CNC, these signals were resolved at peaks 533 and 534 eV, due to the presence of S atoms [38]. Due to this shift, the S-O bond is located at a binding energy of 169 eV for S2p.

Table 4 shows the atomic composition of soluble-grade cellulose pulp and CNC-S. After hydrolysis, the total SO_4_ group content of CNC-S was 4.92 wt%. The stoichiometry of the sample could not be determined, as the H atom was not detected by the XPS technique. However, the total percentage of O atoms was higher compared to soluble-grade cellulose due to an increase in OH bond formation.

The values obtained in the analysis of soluble-grade cellulose pulp (α-cellulose) are similar to the results depicted at the database of the natural polymers manual [42], in where two components for C1s are reported, with binding energies of 286.73 and 288.06 eV for the C-C and C-O bonds, respectively. For O1s spectra, two components at 532.93 eV for the O-H bond, and 533.51 eV for the C-O are shown.

### 3.4. Fourier-Transform Infrared Spectroscopy (FTIR)

**Figure 4:**
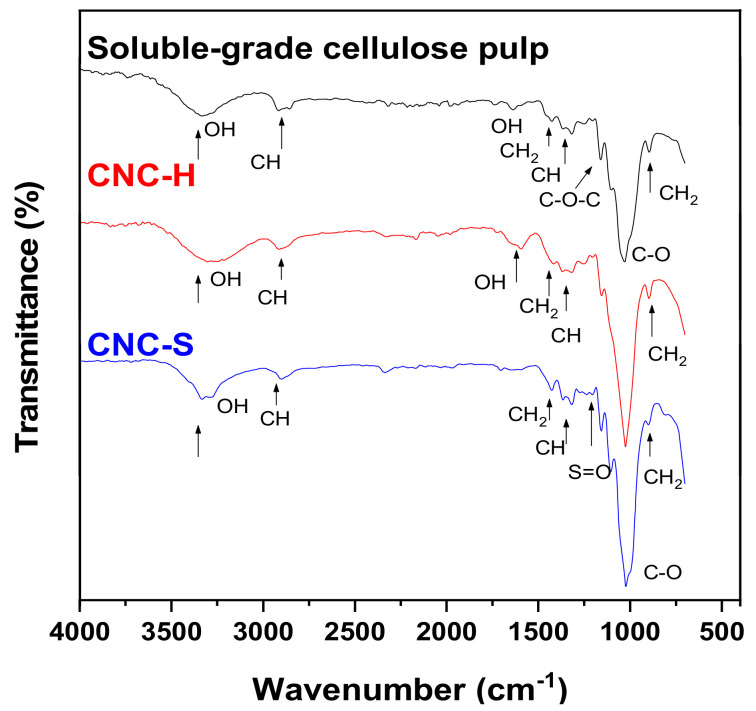
FTIR spectra comparison for soluble-grade cellulose pulp, CNC-H (E10H), and CNC-S (E3S).

Figure 4 shows the comparison of the spectra of soluble-grade cellulose pulp and CNC-S (E10S) and CNC-H (E3H). The obtained spectra showed characteristic frequencies of cellulose, where the vibration bands of C–H bonds (2918 cm^−1^ [asymmetric vibrations], 2851 cm^−1^ [symmetric vibrations]), and C–H bonds (1360 and 1318 cm^−1^ [crystal band] [43,44]) were observed, the latter being an indicator of the presence of CNC, as the band is associated with the preferential directional arrangement of α-cellulose. Bands corresponding to C–O bonds (1054 and 1030 cm^−1^) were observed, whereas those at frequencies of 1160 cm^−1^ were due to asymmetric vibrations (C–O–C bonds), and O–H bonds (broadband between 3600 and 3200 cm^−1^, as well as bands at 1335 and 1205 cm^−1^). The vibration of the anomeric carbon group of the carbohydrate C^1^–H is observed at 898 cm^−1^ and the band corresponding to adsorbed water is observed at 1635 cm^−1^ [13,45,46].

The CNC-S spectrum showed a weak sulfur peak at 1202 cm^−1^, which does not appear in the FTIR spectrum of soluble-grade cellulose pulp. This peak is associated with S=O bonds, indicating esterification of the hydroxyl group during acid hydrolysis. On the other hand, a band corresponding to chlorine ions was not observed for CNC-H, indicating that the hydrolysis with HCl resulted in hydroxyl groups, which are also present in the soluble-grade cellulose pulp [20,22]. The less intense peak located in the region between 3400 and 3200 cm^−1^ indicated the crystallinity of both CNC, since the hydrogen bond is less flexible in the crystalline structure [23,47,48].

The sensitive region in FTIR for the detection of the crystallinity of cellulosic materials was located between 850 and 1500 cm^−1^ [47]. The order index is defined by Oconor et al. [47] as the absorbance ratio of the bands detected at wavelengths of 6.9 and 11 μ (1430 and 900 cm^−1^, known as crystalline and amorphous bands of cellulose) corresponding to vibrations of CH_2_ (the symmetric and rolling bending, respectively) and is defined as LOI: A_1430_/A_900_ [47,49,50,51,52,53,54]. The absorbance ratio of 1363 and 2907 cm^−1^ was attributed to the flexural and stretching vibrations of C-H bonds. This relationship is known as the total crystallinity index (TCI: A_1363_/A_2907_) [51,54,55,56]. The hydrogen bonding intensity is reported by several authors [49,50,52,57] as the ratio A_3350_/A_1337_. The lateral (LOI) and total (TCI) crystallinity indices, as well as the hydrogen bond intensity (HBI) for soluble-grade cellulose, were 1.04, 1.06, and 0.87, respectively. As a reference, the values for Whatman paper were 1.21, 0.89, and 0.99, respectively. For CNC-S and CNC-H samples (E1S, E8S, E10S, E3H, and E8H), LOI and TCI values of 1.03 ± 0.17 and 1.00 ± 0.13 were obtained, similar to those of soluble cellulose. In contrast, the obtained HBI values (1.14 ± 0.1) were slightly higher than that of soluble-grade cellulose pulp. These values were not related to acid nor hydrolysis conditions and were different from those reported for CNC-S obtained from sugar cane bagasse (LOI: 0.57, TCI: 1.32) [51].

### 3.5. X-ray Diffraction (XRD)

**Figure 5:**
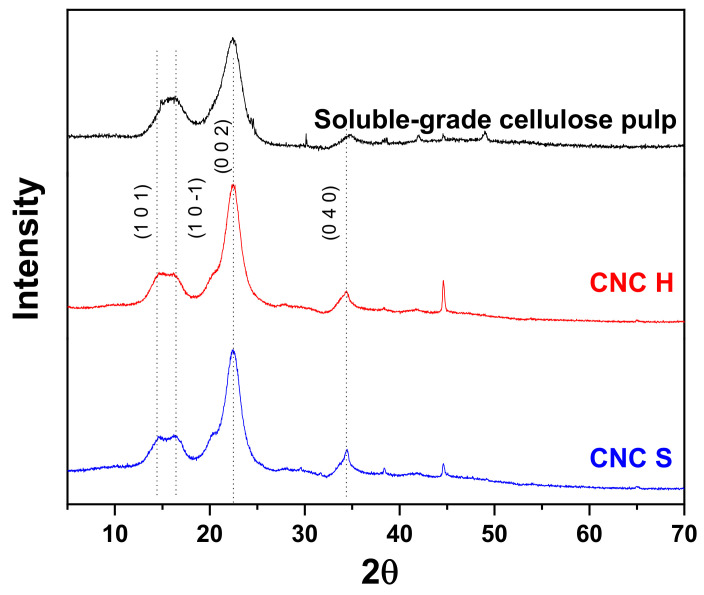
XRD spectra comparison for soluble-grade cellulose pulp, CNC-H (E10H), and CNC-S (E3S).

Figure 5 shows the diffractograms of the soluble-grade cellulose pulp, CNC-H, and CNC-S. Peaks at 2θ angles for the three spectra are similar to that reported in the literature for cellulose crystalline allomorphs [13] with main signals at 2θ = 14.5° (1 0 1), 16.5° (1 0 −1), 22.4° (0 0 2) and 34.8° (0 4 0) [58]. The curves showed peaks associated with cellulose corresponding to the crystalline planes, which are present in all cases with different intensity and broadening. A very common assumption is that the increased amorphous region contribution is the main source of peak broadening [44]. However, several factors may influence peak broadenings, such as anisotropy and nanocrystal size [59]. This could explain the peak at 22.4° for CNC-S and CNC-H, where a slight broadening can be observed at the beginning, which is not observed in the spectra of soluble-grade cellulose pulp.

The crystallinity percentages were calculated with the Rietveld method [40] (Figure S2). The crystallinity values for CNC were recorded for samples E1S (88.4%), E8S (91.3%), and E10S (89.7%), whereas the crystallinity value for CNC-H was recorded for sample E3H (90.1%). Compared to a previous work developed by our research group, a crystallinity value of 79.2% was obtained from soluble-grade cellulose pulp [27]. The values herein reported are higher than those found in the literature for Avicel^®^ PH-102 (51%) [60], Kraft eucalyptus dry lap pulp (73%, H_2_SO_4_ concentration of 58 wt.%) [61], and *A. tequilana* (TEMPO/NaOCl/NaBr system, 78.5%) [62].

### 3.6. Atomic Force Microscopy (AFM)

**Figure 6:**
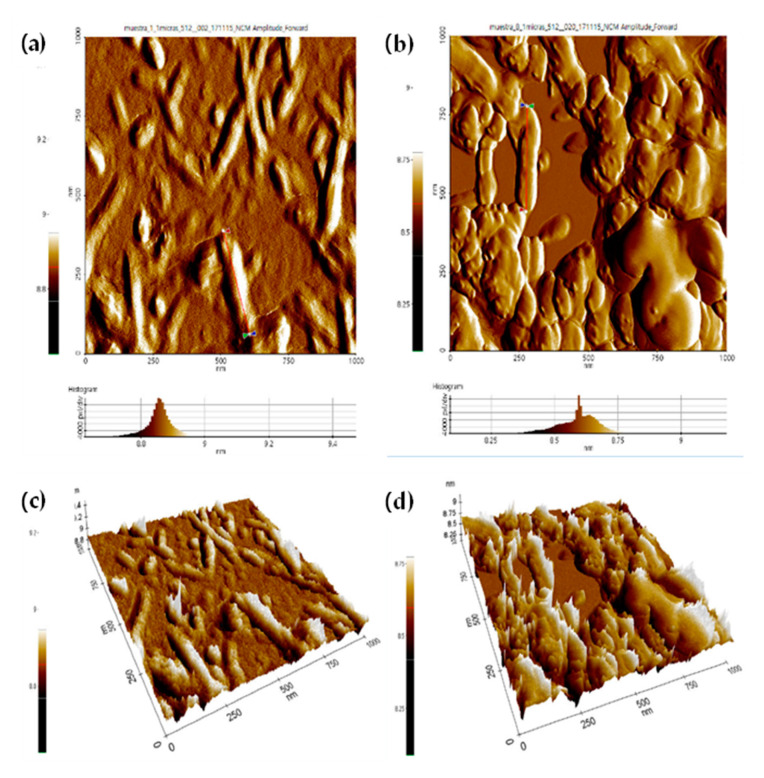
AFM images of CNC-H for two different conditions: (**a**) 2D image and histogram of E1H, (**b**) 2D image and histogram of E8H, (**c**) 3D image of E1H, (**d**) 3D image of E8H.

AFM measurements were carried out to measure the length (L), height (H), and width (W) of dry-deposited CNC-H and CNC-S. AFM images of CNC-H are shown in 2D and height histogram for samples E1H (Figure 6a) and E8H (Figure 6b), respectively, and their 3D projection (Figure 6c,d, respectively). Long, wide, and non-homogenous crystals were observed for the mildest (E1H) and more severe (E8H) hydrolysis conditions. An average L of 216 ± 73 and 266 ± 107 nm, H of 8.9 and 8.6 nm, and W of 69 ± 17 and 88 ± 15 were measured for E1H (mildest condition) and E8H (severe condition), respectively. For the mildest conditions, CNC-H was thinner and shorter than those obtained with the most severe conditions, but with similar H values.

For the rest of the CNC-H samples, L diminishes as HCl concentration increases, with a minimum and maximum L value of 143 and 1100 nm, respectively. In the case of the width, most of the samples exhibit W values between 52 and 320 nm, and H values of 8.6 and 9.1 nm. The L/H ratios for CNC-H obtained with 2N HCl tend to increase with temperature and time, with values ranging from 20 to 98 nm, whereas L/H aspect ratio values for 8N HCl are in the range of 30 to 40, except for the E2H sample. Finally, L/W ratios are between 2.5 to 20, which have a broader range of values due to the different sizes obtained for the CNC-H. The complete set of AFM images of CNC-H are shown in Figure S3 and the average dimensions obtained at different concentrations, temperatures, and hydrolysis times, as well as aspect ratios, are presented in Table S4. A comparison of CNC-H with different raw materials is shown in Table 5. To our knowledge, there is no information regarding the production of CNC-H from *A. tequilana* Weber var. Azul. The L/D aspect ratio reported in this table is based on the CNC-H height (L/H).

When W and H values are similar, CNC have a cylindrical shape with a circular cross-section. However, our results showed that the cross-section of the nanocrystals is wider than higher, which may correspond to an ellipsoidal shape. In some samples, different W were observed due to the agglomeration of the deposited CNC, but only not-aggregated CNC-H were selected to study L, W, and H measurements. The low stability and agglomeration of CNC-H are expected due to the absence of groups on the surface of these nanocrystals [32].

**Figure 7:**
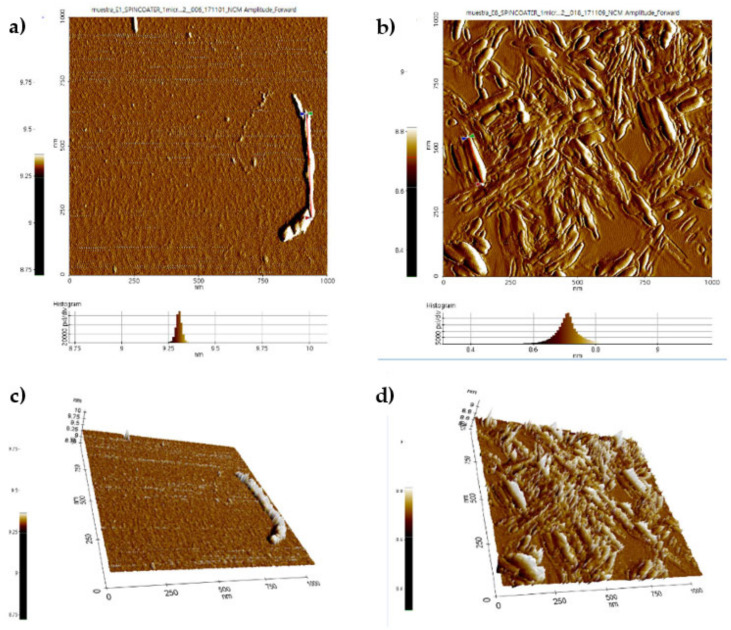
AFM images of CNC-S for two different conditions: (**a**) 2D image and histogram of E1S, (**b**) 2D image and histogram of E8S, (**c**) 3D image of E1S, (**d**) 3D image of E8S.

AFM images of CNC-S for 2D and height histogram for E1S and E8S (Figure 7a,b), and their 3D projection (Figure 7c,d) showed the presence of thin, shorter than CNC-H, ellipsoidal nanocrystals, with an average L of 404 ± 30 and 149 ± 59 nm, H of 9.3 and 9.2 nm, and W of 37 ± 5 and 39 ± 12 measured for E1S (mildest condition) and E8S (severe condition), respectively.

For the rest of the CNC-S samples, the L diminishes as H_2_SO_4_ concentration increases, with a minimum and maximum L value of 88 and 516 nm, respectively. Most of the samples exhibited W values between 20 and 84 nm, and H values of 9.0 and 9.3 nm. The complete set of AFM images of CNC-S are shown in Figure S4 and the AFM dimensions obtained from AFM images are presented in Table S5.

The CNC-S morphology is affected by hydrolysis conditions, and the average L and W decreased with increasing hydrolysis time in the hydrolysis process. As hydrolysis time increased, thinner nanocrystals were observed, regardless of acid concentration or temperature. The recorded W values were 73 ± 11 nm, 49 ± 18 nm, and 33 ± 7 nm at 40, 55, and 70 min for samples E2S, E10S, and E6S, respectively (Figure S4). A similar pattern was also observed for samples E1S, E9S, and E5S. Increasing temperatures from 40 to 60 °C diminished W values from 49 ± 18 to 35 ± 6 nm for E10S and E12S, respectively. The W/H aspect ratio decreased with increasing hydrolysis time, with an average of 4 ± 1 (except for the E2S sample, W/H=8). L/W aspect ratios had an average between 3.7 and 13, and tend to decrease with higher acid concentrations. L/H aspect ratios had a value between 16–43 and, tend to increase with lower acid concentration, time, and temperatures (Table S5).

The L/D aspect ratio reported in Table 6 is based on the CNC-S height (L/H). For most cellulosic nanomaterials, an L/D ≥20 is mentioned [64]. Some of the obtained CNC-S in this work fit into the category of cellulosic nanomaterials. Different CNC-S morphologies obtained from cotton by hydrolysis with H_2_SO_4_ have been reported by Lin and Dufresne (2014) [31], which include circular and ellipsoidal cylindrical shapes as well as flat or laminar shapes. Our results showed that the cross-section of the CNC-S is wider than its high (W > H), which corresponds to an ellipsoidal shape. The dimensions changed with hydrolysis time, temperature, and acid concentration, therefore the change in morphology could be linked to the total sulfate groups present in the CNC-S surface. As the content of total sulfate groups in the CNC-S increases, W decreases. The increase in surface groups could avoid interactions between nanocrystals and reduce CNC-S agglomerations.

CNC-S with aspect ratios between 10 to 45 have proven useful for their use as reinforcement materials [16]. Comparing to CNC-S obtained in this work with CNC-S obtained from different natural fibers and raw materials (Table 6), we have observed that tuning the dimensions of the final CNC-S depends solely on the process parameters, rather than on the material origin.

### 3.7. Zeta Potential, Dynamic Light Scattering (DLS), and Atomic Force Microscopy (AFM) Comparison

To determine the stability of the CNC solution, Z-potential measurements were performed as an indicator of the degree of electrostatic repulsion between the charges of the dispersion particles. Particles with absolute values in the 40–60 mV range showed stability, while those with absolute values lower than 30 mV tend to aggregate [66]. The average Z-potential of CNC-S (E8S) showed values of −54.1 ± 6.2 mV, which is considered stable, due to the presence of negative sulfate groups on the surface. For CNC-H (E8H), the average Z potential value was −25.1 ± 0.89 mV. CNC-H had lower stability than CNC-S due to the presence of OH groups on the surface, as observed by AFM. Thus, CNC-H obtained by HCl hydrolysis show a greater predisposition to agglomerate (Figure 6 and Figure S3). The average Z-potential for the 12 CNC-H samples was −23.5 ± 3 mV. In the case of CNC-S at a hydrolysis temperature of 40 °C, the Z-potential values were <30 mV for two acid concentrations (60 and 65%). In contrast, at hydrolysis temperature of 60 °C, the absolute values of Z-potential were >30 mV. The complete list of values for Z potentials are reported in Table S3.

The CNC particle size obtained by DLS was compared to the L values obtained by AFM, for both acid hydrolysis. The CNC morphology and aspect ratio are also discussed as a function of acid and hydrolysis conditions. The complete list of values of the hydrodynamic diameter (d) and PdI are shown in Tables S1 and S2.

**Figure 8:**
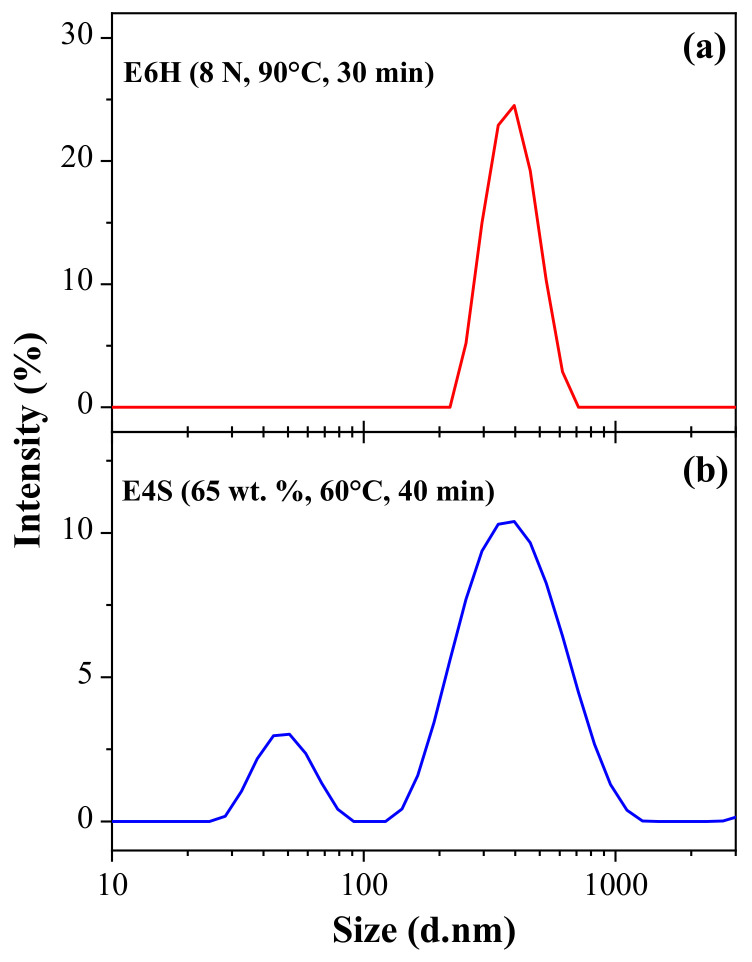
Representative CNC particle size distribution by DLS: (**a**) CNC-H (E6H) y (**b**) CNC-S (E4S).

Figure 8a shows the particle size distribution obtained by DLS for CNC-H corresponding to sample E6H. The rest of CNC-H samples showed a similar size distribution behavior, which corresponded to a Gaussian type, with a unimodal distribution. For these samples, the polydispersity index (PdI) was on average 0.32 ± 0.09. The smallest value was obtained for E12H sample with a hydrodynamic diameter of 323 ± 18 nm and a PdI of 0.24 ± 0.06, while the highest value was observed for E2H sample with a hydrodynamic diameter of 794 ± 245 nm and a PdI of 0.50 ± 0.22.

The size distributions of the CNC-S showed a monomodal distribution at the lowest limit of temperature (40 °C) and time (40 min) for both acid concentrations (60 and 65 wt%, experimental conditions E1S and E2S). The samples showed PDI values of 0.31 and 0.36 for ES1 and ES2, respectively. The experimental setup E4S changed to a bimodal size distribution (Figure 8b), in which two distinctive peaks of 47 ± 16 nm (14 ± 5%), and 370 ± 109 nm (78.7 ± 7%) were observed for the hydrodynamic diameters. In almost all CNC-S samples, the first peak represented 10–40% of the signal intensity, with particle sizes between 30 and 100 nm, while the second peak represented 60–90% of the intensity distribution, with particle sizes between 200–550 nm. Samples are bimodal with PDI average values above 0.66 ± 0.12 [67].

Similar bimodal size distributions were also observed for CNC-S obtained from laser printer paper [68]. The bimodal distribution was due to the presence of different fiber sizes in the paper, and the selected parameters to obtain CNC. In contrast, the bimodal size distribution observed for CNC-H and CNC-S from *Acacia farneciana* L. Willd was attributed to the bleaching process and specific hydrolysis conditions [32]. On the other hand, diprotic acids such as H_2_SO_4_ produce monovalent and divalent ions (HSO_4_^−^ and SO_4_^=^), and the composition in aqueous systems depends on the concentration and temperature of the system. This suggests that during hydrolysis, both HSO_4_^−^ and SO_4_^=^ ions coexist, which probably can attack pulp in different ways, generating nanocrystals with varied morphologies and dimensions.

**Figure 9:**
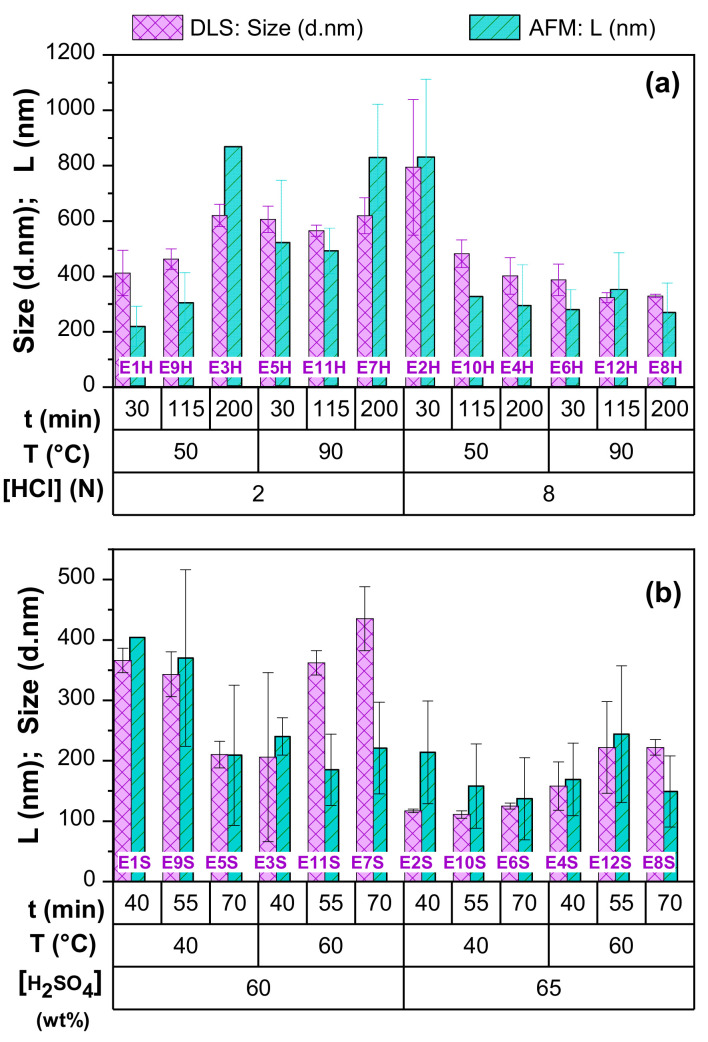
CNC length comparison measured using AFM and DLS for both acid hydrolysis: (**a**) CNC-H (**b**) CNC-S.

Figure 9 shows a comparison of the average particle size of the CNC obtained by DLS and AFM. Larger CNC-H particle sizes were obtained (Figure 9a, 300–800 nm), compared to CNC-S (Figure 9b, 100–500 nm).

The recorded L values measured with AFM were relatively similar to the hydrodynamic diameters (d) measured by DLS. As DLS is faster than AFM, it is usually the preferred technique to estimate CNC lengths obtained from different types of raw materials.

HCl concentration was a determining factor in the size of the nanocrystals, obtaining smaller sizes when concentrations were higher (8N) (Figure 9a). In this case, the hydrodynamic diameters oscillated between 300–500 nm for HCl 8 N, except for the lower limits of temperature (50 °C) and hydrolysis time (30 min) where the diameter is 829 ± 282 nm. Lower concentrations of HCl (2N) gave higher hydrodynamic diameters of 450–800 nm. Similarly, CNC-H diameter did not rely on temperature at low concentrations of HCl, with an average size of 570 ± 164 nm. In contrast, at higher HCl concentrations, the size decreased with increasing temperature (50 and 90 °C) and hydrolysis time. Hydrolysis with H_2_SO_4_ gave smaller hydrodynamic diameters than those obtained with HCl (Figure 9b). The size values reported in this graph corresponded to the average size from their bimodal distribution, which is similar to the longer length of elongated ellipsoids, as demonstrated by AFM

**Figure 10:**
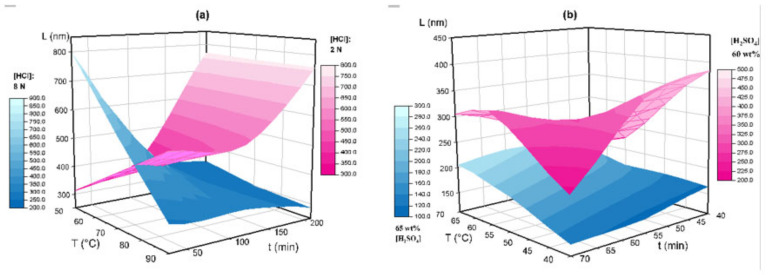
3D representation of lengths measured by AFM for temperature, time, and different acid conditions: (**a**) HCl hydrolysis, 8N (blue), and 2N (magenta); (**b**) H2SO4 hydrolysis, 65 wt% (blue) and 60 wt% (magenta).

Figure 10 shows the effect of hydrolysis conditions with HCl (Figure 10a), and H_2_SO_4_ 60 and 65 wt% (Figure 10b) on the CNC length measured with AFM.

**Figure 11:**
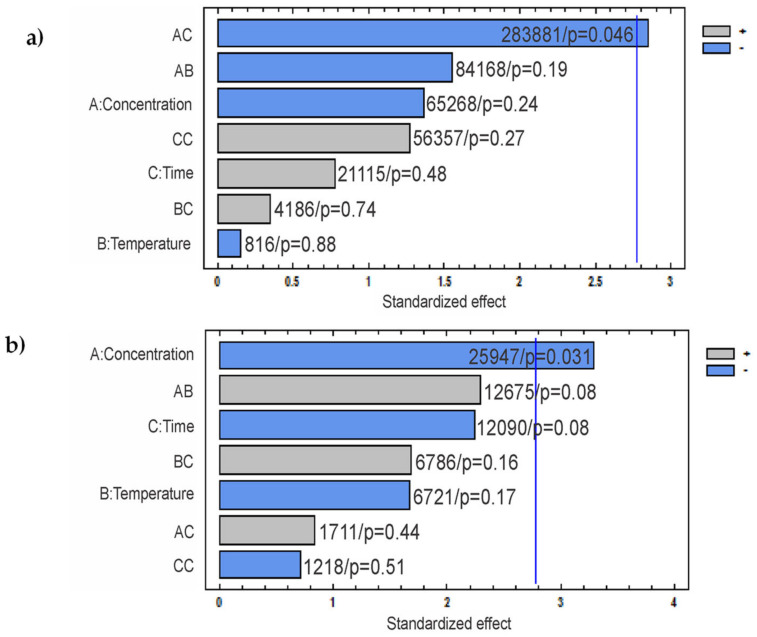
(**a**) Pareto standardized effect for CNC-H length measured by AFM; (**b**) Pareto standardized effect for CNC-S length measured by AFM. The sum of squares and *p*-value is shown at the right side of each bar.

In Figure 10a, smaller CNC-H can be obtained at higher acid concentrations (8 N), with L values between 270–350 nm, except for E2H (829 ± 282 nm). For lower HCl concentrations (2N), CNC-H length values increased with hydrolysis time (from 200 to 800 nm). A correlation with temperature was not observed. The Pareto analysis (Figure 11a) revealed that concentration and time (AC) was the only statistically significant negative interaction reflecting an inverse relationship, which was also visualized in the 3D representation.

The recorded L for CNC-S (Figure 10b) were located between two surfaces, delimited by acid concentration. For 65 wt% H_2_SO_4_, L were in the range of 140–250 nm, while for lower concentrations, L values were in the range of 200–400 nm. A non-linear dependence with time and temperature was also observed. The Pareto analysis (Figure 11b) showed that the concentration was the only statistically significant factor contributing to CNC-S length.

## 4. Future Research Direction

In this work, *Agave tequilana* Weber var. azul bagasse, an agro-industrial waste from the tequila production, represents an alternative, low-cost cellulose source for the formation of CNC. CNC are excellent candidates for their use as composites materials, and low amounts of CNC can improve the final mechanical properties. For example,0.5 wt% of CNC-S in PLA polymeric matrices increased by 65% and 25% the maximum tensile stress and Young’s modulus, respectively [69]. In cementitious matrices, the compressive strength increased by 32% using 0.35 wt% CNC-S or 0.5% CNC-H. The flexural strength value increased by 22% and 17% for 0.5 wt% CNC-S and 0.5 wt% CNC-H, respectively [70]. The potential use of CNC in cementitious matrices is a novel and promising research area, as there is still scarce information in the literature.

## 5. Conclusions

- CNC were successfully obtained from soluble-grade cellulose pulp from *A. tequilana* Weber var. Azul bagasse using an experimental design 2^3^ for two acids, H_2_SO_4_ and HCL.
- The maximum CNC yield was 90 and 96% for CNC-H and CNC-S, respectively, for the most severe hydrolysis conditions tested.
- For CNC-S, the total sulfate group content measured followed an exponential behavior as a function of time: $C_{SG}=ae^{bt}$. The *C_SG_* increased with hydrolysis time, temperature, and H_2_SO_4_ concentration. The *C_SG_* values ranged from 10 to 150 mmol/kg, depending on the hydrolysis conditions.
- The insertion of sulfate groups on CNC-S was also corroborated using XPS, and the results showed 4.92% of sulfate groups for E8S. The presence of sulfate groups on CNC-S was also detected by FTIR.
- The FTIR spectra showed LOI and TOI similar to those of soluble-grade cellulose pulp, near to 1 in both cases (1.03 ± 0.17 and 1.00 ± 0.13 respectively), while HBI increased about 30%.
- The CNC crystallinity was obtained by XRD using the Rietveld method. For all the analyzed samples, the crystallinity values ranged from 88.4 to 91.3%, while the value for soluble grade cellulose pulp was 79.2%
- The Pareto analysis revealed that HCl concentration and time factor interaction (AC) is statistically significant on CNC-H length, whereas H_2_SO_4_ concentration is the only statistically significant factor for CNC-S length.
- The CNC lengths obtained by AFM are very similar to the diameter (d) obtained using DLS.
- The CNC-S lengths were shorter and thinner than those of CNC-H, probably attributed to sulfate group insertion and less agglomeration of the nanocrystals in dispersion.
- The smallest CNC-H length was observed with 8N HCl (266–350 nm, except for sample E2H), and the longest CNC-H was obtained with 2N HCl (256–867 nm). The smallest CNC-S length was obtained with 65 wt% H_2_SO_4_ (137–244 nm) and the longest CNC-S was obtained with 60 wt.% H_2_SO_4_ (185–404 nm).
- As the obtained CNC have an ellipsoidal shape, the studied dimensions were L, H, and W. For both acids (H2SO4 and HCl), CNC height ranged between 8.6–9.3 nm, whereas the width ranged between 20–85 nm, and 47–300 nm, for CNC-S, and CNC-H, respectively. Considering that 2 of 3 dimensions are in the nanometer range, the CNC obtained in this work can be considered as nanomaterials.
- Owing to their exceptional mechanical properties, CNC are excellent candidates to be used as reinforcement materials.

## Figures and Tables

**Table 1:** Controllable process factors and their respective levels for CNC acid hydrolysis length optimization.

	H_2_SO_4_			HCl		
	−1	0	+1	−1	0	+1
**Concentration**	60 wt%	-	65 wt%	2 N	-	8 N
**Temperature (°C)**	40	-	60	50	-	90
**Time (min)**	40	55	70	30	115	200

**Table 2:** Multilevel factorial design 2^3^ process factors for CNC acid hydrolysis length optimization.

H_2_SO_4_				HCl			
Sample	Conc. (wt%)	T (°C)	t (min)	Sample	Conc. (N)	T (°C)	t (min)
**E1S**	60	40	40	**E1H**	2	50	30
**E2S**	65	40	40	**E2H**	8	50	30
**E3S**	60	60	40	**E3H**	2	50	200
**E4S**	65	60	40	**E4H**	8	50	200
**E5S**	60	40	70	**E5H**	2	90	30
**E6S**	65	40	70	**E6H**	8	90	30
**E7S**	60	60	70	**E7H**	2	90	200
**E8S**	65	60	70	**E8H**	8	90	200
**E9S**	60	40	55	**E9H**	2	50	115
**E10S**	65	40	55	**E10H**	8	50	115
**E11S**	60	60	55	**E11H**	2	90	115
**E12S**	65	60	55	**E12H**	8	90	115

**Table 3:** Total sulfate groups content equation parameters.

T(ºC) [H_2_SO_4_] (wt%)	40			60		
	*a* (*mmol/kg*)	b (*1/min*)	R^2^	*a* (*mmol/kg*)	b (*1/min*)	R^2^
**60**	1.90 ± 0.84	0.04 ± 8 × 10^−3^	0.8976	1.46 ± 0.69	0.07 ± 8 × 10^−3^	0.9446
**65**	1.95 ± 0.13	0.01 ± 1 × 10^−3^	0.9983	5.38 ± 0.015	0.05 ± 5 × 10^−5^	1.0

**Table 4:** XPS atomic composition of soluble-grade cellulose pulp and CNC-S (E8S).

Sample Z		Soluble-Grade Cellulose Pulp			CNC (E1S)		
		C	Atom %	Atom %	C	Atom %	Atom %
**C1s**	C-O-C	4.51	10.96	57.82	5.63	7.58	56.12
	C-O, C-OH	12.89	31.32		23.58	31.72	
	C-C	6.40	15.54		12.50	16.82	
**O1s**	O-H	15.44	37.49	42.18	25.74	34.63	38.96
	O-C	1.93	4.69		1.90	2.56	
	O-S	-	-		1.31	1.77	
**S2p**	S-O	41.17	100	0	3.66	4.92	4.92
**Total**		41.17	100	100	74.33	100	100

**Table 5:** Length and diameter comparison of CNC-H from different raw sources.

Source	Conditions	Diameter (nm)	Length (nm)	L/D	Reference
***Agave tequilana*** **Weber var. azul bagasse**	All E1H (2N, 50 °C, 50 min) E8H (8N, 90 °C, 115 min)	8.6–9.1 8.9 8.6	216–829 216 266	29.9–95.2 29.9 30.9	This work
**MCC**	4N, 80 °C, 225 min	10–20	-	-	[63]
**MCC**	8N, 110 °C, 180 min	14–16	200–250	14–15	[13]
**MCC**	6N, 110 °C, 180 min	10–30	190–250	10–25	[22]
***Acacia farnesiana* L. Willd**	2–8 N, 50–90 °C, 30–200 min	-	100–512	-	[32]

**Table 6:** Dimension comparison of CNC-S from different raw sources.

Source	Conditions	Diameter (nm)	Length (nm)	L/D	Reference
***Agave*** ***tequilana*** **Weber var. Azul**	All E1S (60 wt%, 40 °C, 40 min) E8S (65 wt% N, 60 °C, 70 min)	8.7–9.3 9.3 9.2	137–404 404 149	14.7–44.4 43.4 16.2	This work
***Agave*** ***angustifolia***	60 wt%, 45 °C, 45 min	8–15	170–500	10–45	[17]
***Agave*** ***sisalana***	55 wt%, 45–60 °C, 20–30 min	5.9–10.5	177–433	14–15	[65]
***Agave*** ***tequilana***	65 wt%, 50 °C, 60 min	11	323 ± 112	28	[15]
**Barley**	65 wt%, 50 °C, 60 min	10	329 ± 123	32	[15]
***MCC***	64 wt%, 44 °C, 130 min	16	218 ± 56	13	[15]
***MCC***	50 °C, 60 min	14–16	200–250	14–15	[13]
***Acacia farnesiana* L. Willd**	60–65 wt%, 45–55 °C, 45–65 min	–	100–260	–	[32]

## Data Availability

The data that supports the findings of this study are available from the corresponding author, E.R.M.-B., upon reasonable request.

## Supplementary Materials

The following are available online at https://www.mdpi.com/2079-4991/11/2/520/s1, Figure S1. (a) Pareto standardized effect for CNC-H yield production; (b) Pareto standardized effect for CNC-S yield production. The sum of squares and *p*-value is shown at the right side of each bar; Figure S2. Rietveld analysis for (a) CNC-S (E3S), (b) CNC-H (E10H); Figure S3. AFM 2D images of the 12 CNC-H samples; Figure S4. AFM 2D images of the 12 CNC-S samples; Table S1. DLS and yield results for CNC-H production; Table S2. DLS and yield results for CNC-S production; Table S3. Total sulfate groups of CNC-S; Table S4. AFM measurements of CNC-H; Table S5. AFM measurements of CNC-S.
