# Supplementary material for: Optimization of the Obtaining of Cellulose Nanocrystals from Agave tequilana Weber Var. Azul Bagasse by Acid Hydrolysis
Source: Nanomaterials (Basel). 2021 Feb 18;11(2):520. doi: 10.3390/nano11020520 (PMC7922795; doi:10.3390/nano11020520)
Supplement: Supplementary file 1 [file nanomaterials-11-00520-s001.zip › nanomaterials-1106824-supplementary.pdf]

nanomaterials

Supplementary Information

Optimization of the obtaining of cellulose nanocrystals from *Agave tequilana* Weber var. Azul by acid hydrolysis

Manuel Alberto Gallardo-Sánchez [1], Tania Diaz-Vidal [2], Edgar Benjamín Figueroa-Ochoa [3], Rogelio Ramírez Casillas [4], José Anzaldo Hernández [4], Luis Carlos Rosales-Rivera [2], J. Felix Armando Soltero Martínez [2], Salvador García Enríquez [4], Emma Rebeca Macías-Balleza [2,*]

[1] Departamento de Ingeniería de Proyectos, Centro Universitario de Ciencias Exactas e Ingenierías, Universidad de Guadalajara, Guadalajara, Mexico; manuel.gallardo@academicos.udg.mx
[2] Departamento de Ingeniería Química, Centro Universitario de Ciencias Exactas e Ingenierías, Universidad de Guadalajara, Guadalajara, Mexico; taniadzv@hotmail.com, carlos.rosales@academicos,udg.mx, armando.soltero@academicos.udg.mx,
[3] Departamento de Química, Centro Universitario de Ciencias Exactas e Ingenierías, Universidad de Guadalajara, Guadalajara, Mexico; benjamin.figueroa@academicos.udg.mx
4 Departamento de Madera Celulosa y Papel, Centro Universitario de Ciencias Exactas e Ingenierías, Universidad de Guadalajara, Guadalajara, Mexico; roramire@gmail.com, j.anzaldo@academicos.udg.mx, salgaren@hotmail.com

* Correspondence: emma.macias@academicos.udg.mx; Tel.: 52 33 13 78 59 00 (27591).

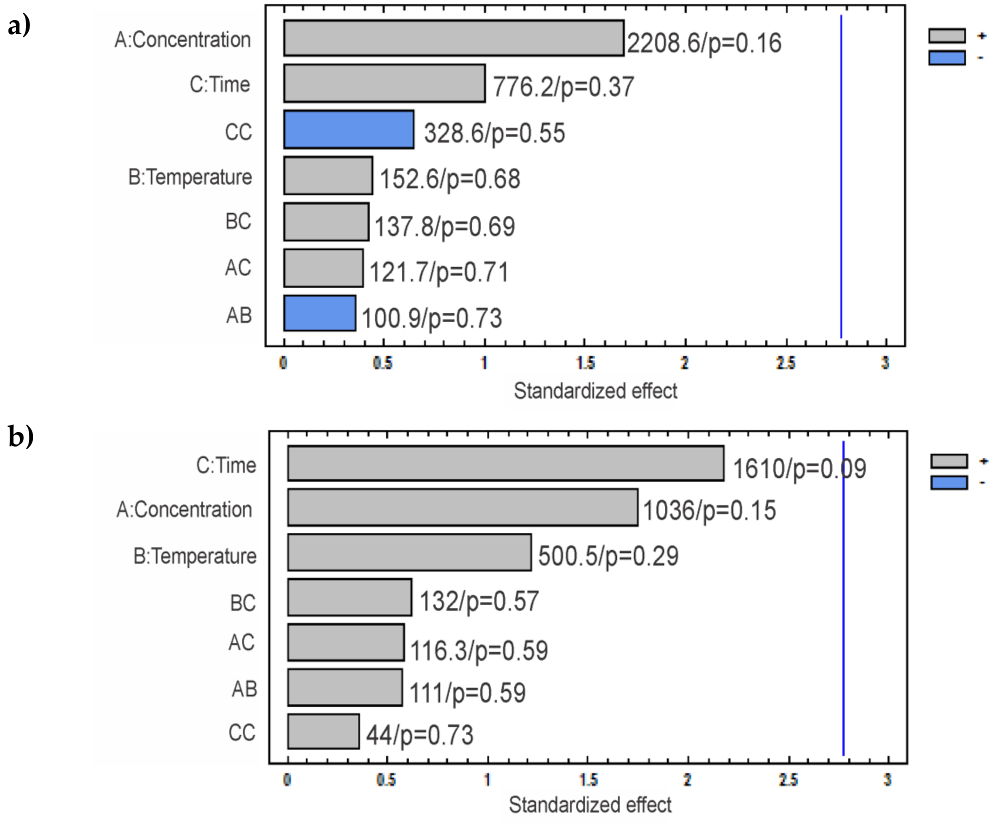

Figure S1. **(a)** Pareto standardized effect for CNC-H yield production; **(b)** Pareto standardized effect for CNC-S yield production. The sum of squares and p-value is shown at the right side of each bar.

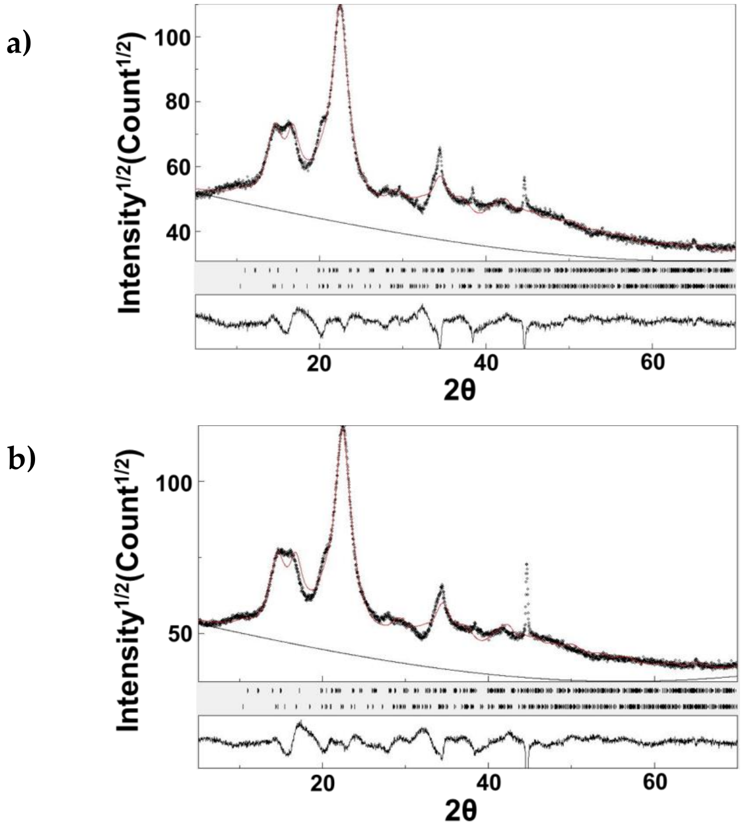

Figure S2. Rietveld analysis for (**a**) CNC-S (E3S), (**b**) CNC-H (E10H)

HCl (N)	T (°C)	30 min	115 min	200 min
2	50	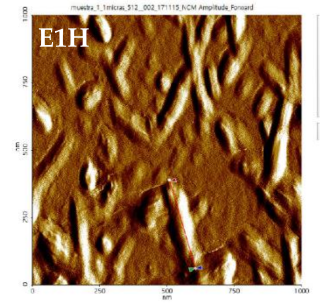	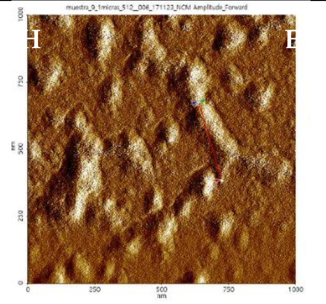	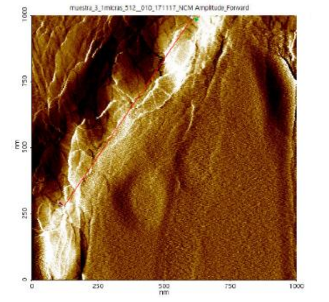
	90	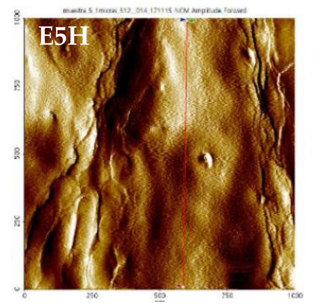	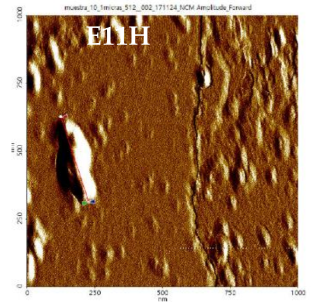	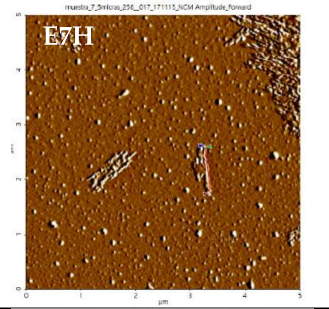
8	50	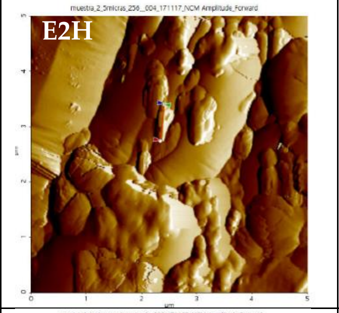	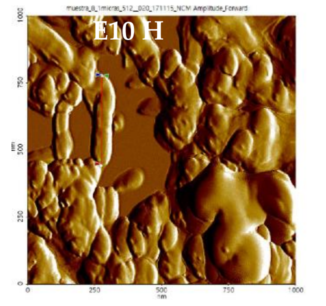	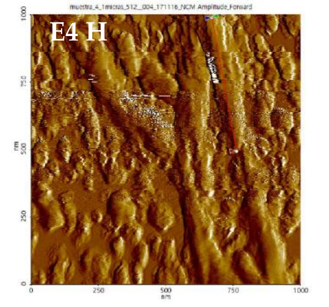
	90	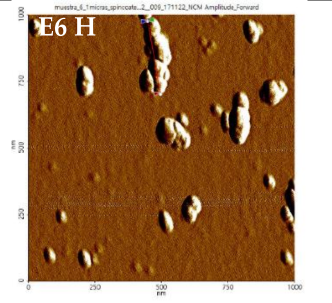	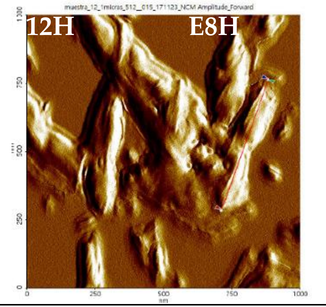	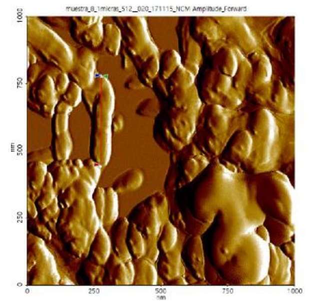

Figure S3. AFM 2D images of the 12 CNC-H samples

H_2SO_4 (wt%)	T (°C)	40 min	55 min	70 min
60	40	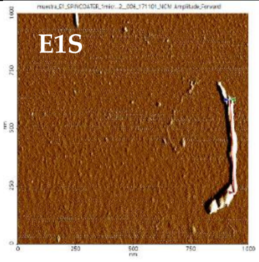	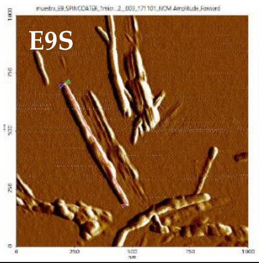	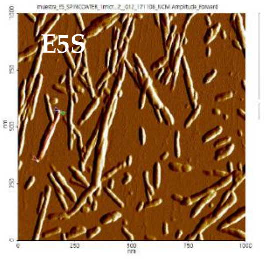
	60	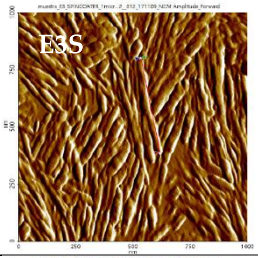	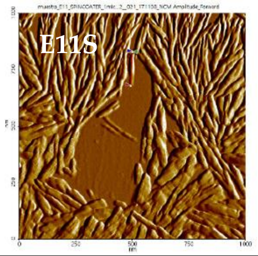	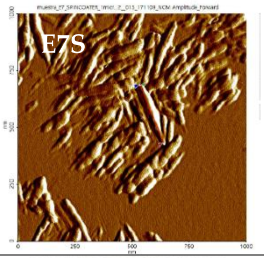
65	40	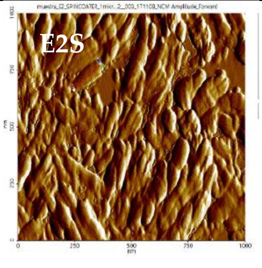	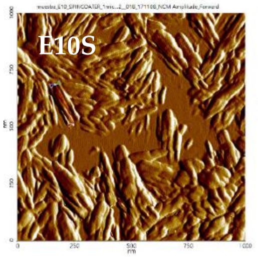	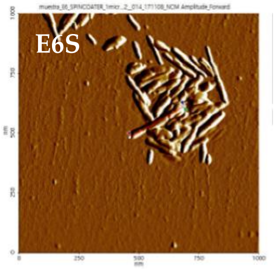
	60	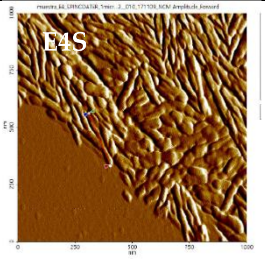	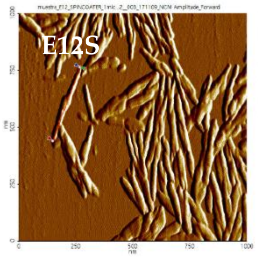	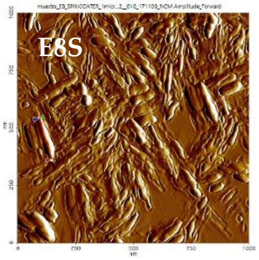

Figure S4. AFM 2D images of the 12 CNC-S samples

Table S1. DLS and yield results for HCl hydrolysis

Hydrolysis Conditions			Sample		DLS			
							Peak 1	
HCl (N)	T (°C)	t (min)	ID	Yield (%)	Size (d.nm)	PdI	Size (d.nm)	%
2	50	30	E1H	4.2	412±82	0.30±0.09	447±87	98±2
		115	E9H	34	462±56	0.32±0.07	499±59	98±2
		200	E3H	22	620±40	0.26±0.05	627±58	98±2
	90	30	E5H	30	606±48	0.27±0.04	738±169	99±1
		115	E11H	33	565±20	0.34±0.06	614±22	100
		200	E7H	36	619±65	0.45±0.07	526±92	88±2
8	50	30	E2H	32	794±245	0.50±0.22	617±231	100
		115	E10H	90	482±50	0.45±0.04	376±62	99±1
		200	E4H	37	402±66	0.34±0.05	437±69	97±2
	90	30	E6H	40	387±87	0.26±0.11	373±58	99±1
		115	E12H	33	323±18	0.24±0.06	322±34	99±1
		200	E8H	90	329±6	0.19±0.05	355±18	100

Table S2. DLS and yield results for H_2SO_4 hydrolysis conditions

Hydrolysis conditions			Sample	Results							
				Yield	[AG]	DLS					
								Peak 1		Peak 2	
H_2SO_4 (wt%)	T (°C)	t (min)	ID	(%)	$\frac{mmol}{Kg}$	Size (d.nm)	PdI	Size (d.nm)	%	Size (d.nm)	%
60	40	40	E1S	4.5	10	366 ± 20	0.31 ±0.06	379 ±201	97 ±2	-	-
		55	E9S	34	15	343 ± 37	0.45 ±0.12	386 ±39	85 ±7	50 ±2	6 ±1.5
		70	E5S	32	35	210 ± 22	0.50 ±0.04	470 ±110	75 ±12	73 ±24	24 ±13
	60	40	E3S	23	15	206 ± 140	0.74 ±0.14	260 ±126	79 ±11	38 ±32	18 ±10
		55	E11S	31	31	362 ± 20	0.85 ±0.21	552 ±41	63 ±8	93 ±22	12 ±0.2
		70	E7S	37	69	435 ±53	0.70 ±0.19	473 ±60	87 ±4	75 ±22	11 ±3
65	40	40	E2S	29	21	117 ±3	0.36 ±0.11	177 ±69	93 ±5	-	-
		55	E10S	37	72	111 ±6	0.58 ±0.05	356 ±125	58 ±6	52 ±10	38 ±10
		70	E6S	42	162	125 ±5	0.61 ±0.12	306 ±141	68 ±11	48 ±17	28 ±10
	60	40	E4S	37	38	158 ±40	0.79 ±0.16	370 ±109	78 ±7	47 ±16	14 ±5
		55	E12S	32	80	222 ±76	0.68 ±0.18	326 ±76	78 ±7	40 ±22	12 ±4
		70	E8S	96	166	222 ±13	0.69 ±0.24	329 ±25	87 ±8	33 ±9	10 ±1.5

Table S3. Total sulfate groups

Experiment	Total Sulfate Groups (mmol/Kg)
**E1S**	**10.34**
**E2S**	**54.77**
**E3S**	**20.73**
**E4S**	**38.18**
**E5S**	**35.32**
**E6S**	**68.85**
**E7S**	**162.01**
**E8S**	**166**
**E9S**	**15.45**
**E10S**	**31.16**
**E11S**	**71.74**
**E12S**	**79.51**

Table S4. AFM measurements of CNC-H

Hydrolysis conditions			Sample	AFM					
				Dimensions			Aspect ratio		
HCl (N)	T (°C)	t (min)	ID	L (nm)	H (nm)	W (nm)	L/H	L/W	W/H
2	50	30	E1H	216 ±73	8.9	69 ± 17	24 ± 8	3.6 ± 1.9	7.8 ± 1.9
		115	E9H	302 ± 109	9.1	77 ± 16	33 ± 12	85 ± 3	8.5 ± 2.9
		200	E3H	867	8.8	-	98	-	-
	90	30	E5H	520 ± 225	8.7	65 ± 7	60 ± 26	8.5 ± 4.4	7.5 ± 0.8
		115	E11H	490 ± 82	9.1	109 ± 24	54 ± 9	4.9 ± 1.8	12 ± 2.6
		200	E7H	828 ± 193	8.7	68 ± 17	95 ± 22	13.7 ± 6.3	7.8 ± 2.6
8	50	30	E2H	829 ± 282	9.1	253 ± 70	91 ± 31	3.9 ± 2.2	28 ± 8
		115	E10H	324	9.1	103 ± 14	36	3.2 ± 0.4	11 ± 1.5
		200	E4H	292 ± 147	9.0	87 ± 24	32 ± 16	4.1 ± 2.8	9.7 ± 2.7
	90	30	E6H	277 ± 72	8.9	58 ± 11	31 ± 8	5.2 ± 2.2	6.5 ± 1.2
		115	E12H	350 ± 133	9.1	103 ± 46	38 ± 14	5 ± 3.5	11 ± 5.1
		200	E8H	266 ± 107	8.6	88 ±15	31 ± 12	3.3 ± 1.8	10 ± 1.7

Table S5. AFM measurements of CNC-S

Hydrolysis conditions			Sample	AFM					
H_2SO_4] (wt%)	T (°C)	t (min)	ID	Dimensions			Aspect ratio		
				L (nm)	H (nm)	W (nm)	L/H	L/W	W/H
60	40	40	E1S	404	9.3	37 ± 5	43	11 ± 1.5	4 ± 0.5
		55	E9S	370 ± 146	9.1	36 ± 6	41 ± 16	13 ± 4.2	4 ± 0.7
		70	E5S	209 ±116	9.0	31 ± 9	23 ± 13	10 ± 4.8	3.5 ± 1
	60	40	E3S	240 ±31	9.0	38 ± 10	27 ± 3	7.3 ± 2.3	4.2 ± 1.1
		55	E11S	185 ±59	9.0	54 ± 13<br>25 ± 5	21 ± 6	4.3 ±1.6<br>9.2 ±3	6 ± 1.4<br>2.8 ± 0.6
		70	E7S	221 ±76	8.9	40 ± 12	25 ± 8	7.4 ±3.2	4.5 ± 1.3
65	40	40	E2S	214 ±85	8.9	73 ± 11	24 ± 9	3.7 ±3 1	8.2 ± 1.2
		55	E10S	158 ±70	8.7	49 ± 18	18 ± 8	4.9 ±2.5	5.6 ± 2.1
		70	E6S	137 ±68	9.1	33 ± 7	26 ± 7	8.8 ± 2.9	3.6 ± 0.8
	60	40	E4S	169 ±60	8.7	44 ± 12	19 ± 7	5.1 ± 2.1	5.1 ± 1.4
		55	E12S	244 ±113	8.7	35 ± 6	28 ± 13	9.1 ± 3.2	4 ± 0.7
		70	E8S	149 ±59	9.2	39 ± 12	16 ± 6	5.3 ± 2.4	4.2 ± 1.3
